# A New ICD-10 Code for Hemorrhagic Myocardial Infarction

**DOI:** 10.1016/j.jacadv.2026.102740

**Published:** 2026-04-17

**Authors:** Ankur Kalra, Dharam J. Kumbhani, Rohan Dharmakumar

**Affiliations:** a Division of Cardiology, Department of Medicine, State University of New York (SUNY), Upstate Medical University, Syracuse, New York, USA; b Division of Cardiology, Department of Medicine, UT Southwestern, Dallas, Texas, USA; c Cardiovascular Imaging Research Center, Medical Imaging Research Institute, Indiana University, School of Medicine, Indianapolis, Indiana, USA

**Keywords:** intramyocardial hemorrhage, hemorrhagic myocardial infarction, STEMI, PCI, ICD-10

**Figure:**
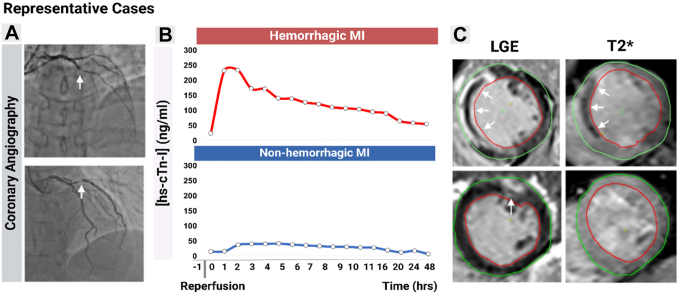

The notion that not all ST-segment elevation myocardial infarctions (STEMIs) are the same is the central tenet of the recently published Canadian Cardiovascular System (CCS) Classification on stages of acute myocardial infarction.^1^ The CCS classification provides a strategy to classify the severity and extent of myocardial injury following reperfused myocardial infarction (MI). A key aspect of the CCS staging is that patient prognosis worsens with progressive stages of tissue injury. Among these, the most severe form of myocardial injury (CCS stage 4) comprises multiple stages of progressive tissue damage with the hallmark injury of intramyocardial hemorrhage (IMH). IMH is a consequence of microvascular rupture, resulting in the extravasation of red blood cells into the interstitial space of the myocardium.

Mechanistic, translational, and clinical studies over the past 2 decades have demonstrated deleterious sequelae of IMH.^2^, ^3^, ^4^ Specifically, mechanistic and translational studies have shown that iron from red blood cells contributes to significant damage in the acute and chronic phases following revascularization. These studies showed that damage from IMH spans extensive loss of salvaged myocardium within the first 24 hours of percutaneous coronary intervention (PCI) to a complex interplay of pathophysiological mechanisms culminating in lipomatous metaplasia of infarcted tissue. Multiple single-center and multicenter clinical studies have repeatedly shown that patients with STEMI and IMH are at multifold higher risk for major adverse cardiovascular events (MACE), including heart failure and death. In fact, some of these studies have shown that microvascular injury carries a 4-fold greater risk of MACE than MI size, a parameter once thought to be the key predictor of post-MI outcomes. Of notable importance is the recent multicenter study reporting outcomes in 1,109 patients with STEMI undergoing primary PCI, which demonstrated that 1-year risk of MACE in patients with hemorrhagic MI was multifold higher than those with microvascular obstruction (MVO) without IMH.^4^ Results of this study are important as: 1) it is the largest longitudinal study using cardiac magnetic resonance imaging (CMR) to assess MACE 1-year post-PCI in patients with STEMI exposed to varying degrees of microvascular injury; 2) it found that when patients are stratified according to MI only, MI with MVO, and MI with MVO plus IMH, the risk of MACE was most significant in those with IMH, but similar in those with or without MVO but no IMH. It is important to note here that MVO broadly defines microvascular injury, but IMH is only found in a subset of patients with MVO; and is never found in the absence of MVO. Hence, the form of microvascular injury, MVO accompanied by microvascular rupture (ie, IMH) compared with MVO alone, transcends the notion that all MVOs are the same. This highlights the importance of distinguishing patients with IMH from those with MVO alone in identifying the most vulnerable patients for post-MI MACE. It is important because without a unique diagnosis code for hemorrhagic MI, STEMI management and outcomes may be hard to both dichotomize based on IMH status and also modify.

The challenge in identifying patients with hemorrhagic MI is that it requires T2∗ CMR, which is difficult to obtain in a timely manner as not all MI patients are suitable for the latter in the acute setting (presence of arrhythmias, difficulty with breath holding, and patient discomfort). In addition, the accessibility of CMR, particularly in community settings, operator expertise, and the need for necessary delay to generate image contrast complicate CMR-based diagnosis of hemorrhagic MI. However, these limitations of CMR-based diagnosis of hemorrhagic MI could be overcome by the recently demonstrated relationship between post-PCI troponin kinetics and IMH.^5^ Troponin assays are universally available in hospitals performing PCI, with results available within hours at minimal additional cost beyond routine post-PCI monitoring. Using separate discovery and validation cohorts, this study demonstrated that it is possible to diagnose patients with hemorrhagic MI within the first 2 hours of PCI from those who are nonhemorrhagic with excellent sensitivity and specificity based on high-sensitivity cardiac troponin I concentration ([hs-cTn-I]) cutoffs.^5^ Figure 1 shows examples of representative cases of patients with and without IMH based on CMR and post-PCI troponin kinetics. Based on post-PCI troponin cutoffs for rapid identification of hemorrhagic MI, it was also shown that patients with hemorrhagic MI are at nearly 3-fold higher risk for in-hospital mortality compared with those without hemorrhagic MI.^5^ Accordingly, based on a growing body of clinical evidence, it is becoming clearer that hemorrhagic MI poses the highest observed risk for adverse outcomes both in the acute and chronic phases of STEMI post-PCI. Yet, because of lack of ubiquity of CMR and variations in use and interpretation of post-PCI troponin kinetics/thresholds, hemorrhagic MI remains underdiagnosed. Other existing limitations and pitfalls for systematically diagnosing hemorrhagic MI include: 1) lack of uniform availability/knowledge of pre-PCI troponin concentration, as continued positive delta is expected in patients with rising troponin before procedure^6^; 2) timing dependency, as the diagnostic performance of troponin thresholds degrades after 10 hours post-PCI (sensitivity >0.84, specificity >0.80, and AUC >0.84 at later timepoints vs >0.91, >0.86, and >0.92, respectively, in the first 10 hours);^5^ 3) current lack of therapeutic interventions to either prevent or treat hemorrhagic MI once identified^7^; and 4) validation limitations as the time-dependent cutoff values were derived and validated in relatively small cohorts (154 discovery, 53 validation) before registry application.^5^

**Figure 1:**
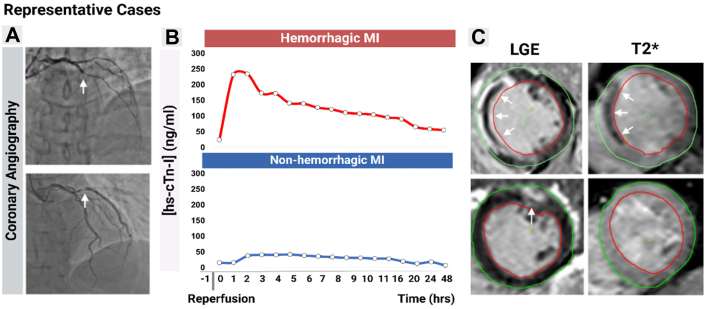
Determining Intramyocardial Hemorrhage With Post-Percutaneous Coronary Intervention Troponin Kinetics and T2∗ Cardiac Magnetic Resonance Imaging Representative cases*:* The top and bottom rows display representative reperfused STEMI patients with and without hemorrhagic MI, respectively. A shows coronary angiograms illustrating the culprit coronary artery occlusions. B shows the differential time-resolved [hs-cTn-I], including pre-PCI, immediately after PCI, and hourly measurements up to 12 hours post-PCI, with additional readings at 20, 24, and 48 hours post-PCI. C shows late-gadolinium-enhanced CMR confirming the presence of MI (arrows) and T2∗ CMR indicating presence (arrows) and absence of IMH. Note that the [hs-cTn-I] threshold for detection of MI is 0.04734 ng/mL, which is significantly smaller compared with [hs-cTn-I] post-PCI in both hemorrhagic and nonhemorrhagic STEMIs but note that the magnitude and pattern of [hs-cTn-I] elevations post-PCI in hemorrhagic MI are significantly different from nonhemorrhagic MI. CMR = cardiac magnetic resonance imaging; IMH = intramyocardial hemorrhage; MI = myocardial infarction; PCI = percutaneous coronary intervention; STEMI = ST-segment elevation myocardial infarction.

One potential path toward systematically defining the epidemiology of hemorrhagic MI in the STEMI population is establishing an ICD-10 code for patients identified with hemorrhagic MI, using a practical approach combining troponin kinetics for initial risk stratification, with CMR reserved for confirmation when clinically indicated. This strategy maximizes diagnostic yield while optimizing resource utilization. A new ICD-10 code will leverage the recent advances to identify the most vulnerable STEMI patients. This has the benefit of enabling close monitoring of patients in the acute care settings prior to discharge and appropriate follow-up postdischarge. It also has the benefit of identifying patients for evolving interventional or pharmaceutical therapies aiming to overcome the development of IMH or mitigate the long-term cardiac toxicity associated with IMH. Given the advances the field of interventional cardiology has made following the introduction of primary PCI as standard of care for STEMI, it is now prudent to start embracing the concept that not all reperfused STEMIs are the same or have similar clinical outcomes. In fact, identifying the most vulnerable patients with STEMI offers an important opportunity to improve their management. Routine assessment of whether a STEMI is hemorrhagic stands to benefit the patient first but could also help triage resources in the acute care settings with the potential to offset the growing costs associated with STEMI care.

## Funding support and author disclosures

Dr Kalra is the founder of makeadent.org. Dr Dharmakumar has received research funding from the 10.13039/100000002NIH (HL133407, HL136578, and HL147133) and has equity interest in Cardio-Theranostics, LLC. Dr Kumbhani has reported that they have no relationships relevant to the contents of this paper to disclose.
